# Cascade Bioassay Evidence for the Existence of Urothelium-Derived Inhibitory Factor in Guinea Pig Urinary Bladder

**DOI:** 10.1371/journal.pone.0103932

**Published:** 2014-08-01

**Authors:** Na N. Guan, Anna Thor, Katarina Hallén, N. Peter Wiklund, Lars E. Gustafsson

**Affiliations:** 1 Department of Physiology and Pharmacology, Karolinska Institutet, Stockholm, Sweden; 2 Section of Urology, Department of Molecular Medicine and Surgery, Karolinska Institutet, Stockholm, Sweden; Cinvestav-IPN, Mexico

## Abstract

Our aim was to investigate whether guinea pig urothelium-derived bioactivities compatible with the existence of urothelium-derived inhibitory factor could be demonstrated by in vitro serial bioassay and whether purinergic P1 receptor agonists, nitric oxide, nitrite or prostaglandins might explain observed activities. In a cascade superfusion system, urothelium-denuded guinea pig ureters were used as bioassay tissues, recording their spontaneous rhythmic contractions in presence of scopolamine. Urothelium-intact or -denuded guinea pig urinary bladders were used as donor tissues, stimulated by intermittent application of carbachol before or during the nitric oxide synthase inhibitor *N^G^*-nitro-L-arginine methyl ester (L-NAME), the adenosine/P1 nucleoside receptor antagonist 8-(*p*-sulfophenyl)theophylline (8-PST) or the cyclo-oxygenase inhibitor diclofenac infused to bath donor and bioassay tissues. The spontaneous contractions of bioassay ureters were unaltered by application of carbachol 1–5 µM in the presence of scopolamine 5–30 µM. When carbachol was applied over the urothelium-denuded bladder, the assay ureter contraction rate was unaltered. Introducing carbachol over the everted urothelium-intact bladder significantly inhibited the contraction frequency of the assay ureter, suggesting the transfer of an inhibitory activity from the bladder to the assay ureter. The transmissible inhibitory activity was not markedly antagonized by L-NAME, 8-PST or diclofenac, while L-NAME nearly abolished nitrite release from the urothelium-intact bladder preparations. We suggest that urothelium-derived inhibitory factor is a transmissible entity over a significant distance as demonstrated in this novel cascade superfusion assay and seems less likely to be nitric oxide, nitrite, an adenosine receptor agonist or subject to inhibition by administration of a cyclo-oxygenase inhibitor.

## Introduction

Lower urinary tract symptoms such as incontinence, urgency and frequent micturitions are prevalent in the older population, where 40% of individuals over age 70 are affected [1]. The primary clinical problem which also has major impact on the patients is urgency to void. The exact mechanisms underlying urgency are currently unclear. The bladder urothelium has long been thought to be a protective barrier between detrusor and urine. In the late 1980’s it was noted that contractile responses to the sensory nerve mediator substance P in the guinea pig urinary bladder were smaller when the urothelium was intact [2]. Later, it was found that in the pig urinary bladder there was an enhanced response to the suggested bladder contractile transmitter substances, and some synthetic analogs, if the urothelium was removed, and that if a second urothelium-intact tissue was co-incubated the responses returned to lower amplitude [3]. Strong evidence for the release of an inhibitory mediator was obtained by co-incubating urothelium-containing urinary bladder with an endothelium-denuded rat aorta strip [4], [5]. This is a sandwich-type bioassay which only demonstrates the transmission of the bioactive principle(s) over a short distance. A cascade superfusion bioassay system would offer further possibilities for pharmacological analysis by physical separation of the tissues, with separate application of modifying or blocking drugs, and if a transmissible bioactivity were to be found could be an advent to isolation of the bioactive principle. The nature of the urothelium dependent inhibitory factor(s) has however not been elucidated. One substance group to be considered is arachidonic acid derivatives from the cyclo-oxygenase system, another being the purine group including adenosine since ATP release is significant in the urothelium [6]–[9]. E-class prostaglandins are usually contractile on bladder detrusor [10], but inhibitory effects have been reported [11]. Experiments in urothelium-intact and -denuded preparations had shown that cyclo-oxygenase products had a role in regulation of ureteral motility [12]. The data suggested that prostacyclin was released from the urothelium, possibly acting via release of an unknown inhibitory factor. ATP released in the bladder and from the urothelium will be metabolized to adenosine [8] which is inhibitory on bladder motility [13], [14] and therefore has to be considered when studying urothelium-derived inhibitory factors. Potent water-soluble adenosine antagonists lacking smooth muscle relaxing effects via phosphodiesterase inhibition have been shown to block inhibitory adenosine receptors in guinea-pig bladder [15] and might be used to explore any involvement of endogenous adenosine.

Early experiments [4], [5], [16], [17] indicated that the inhibitory factor was transmissible within an organ bath, very much in similarity with the first EDRF experiments which led to the discovery of nitric oxide (NO) as a signaling molecule [18]. However, their experiments suggested that the urothelium-derived relaxing factor (UDRF) was not a cyclo-oxygenase product or nitric oxide. In the early experiments on NO, cascade serial superfusion techniques were proven to be much more efficient to demonstrate the release of EDRF and to characterize its half-life and chemical nature [19]. We had utilized this technique in experiments on nitrergic transmission in the gut [20] and presently aimed at investigating whether it may be useful for obtaining further evidence for the existence of urothelium-derived relaxing factor. We also wished to explore whether nitrergic and/or purinergic pathways might be involved. We used carbachol as releasing agonist for the inhibitory factor since this has proven effective in earlier studies and since the urothelium is replete with muscarinic receptors [21].

## Materials and Methods

### Tissue preparations

The experiments were approved by the Stockholm North animal ethics committee (Dnr N173/05, 148/08 and 178/11). Guinea pigs (350–450 g) of either sex were anaesthetized with midazolam+sodium pentobarbital and exsanguinated. The kidneys, ureters and urinary bladders were removed en bloc and the proximal 2 cm of the ureters with at least two thirds of the renal pelvis were isolated. The renal pelvis-ureter preparations were cut open longitudinally, and in some preparations the urothelium was removed by scraping with a syringe needle. The urinary bladders were everted, washed with Tyrode’s solution (136.9 mM NaCl, 4.8 mM KCl, 23.8 mM NaHCO_3_, 0.5 mM MgCl_2_·6H_2_O, 0.4 mM NaH_2_PO_4_·H_2_O, 2.5 mM CaCl_2_, and 5.5 mM glucose) and then tied at both ends with thin cotton threads. In some experiments the bladder urothelium was removed by cutting with scissors. Successful removal of the urothelium from ureters and bladders was checked by staining, see below. All tissues were equilibrated for 60 min in a storage bath with Tyrode’s solution aerated with 5% CO_2_ in O_2_ at 37°C.

### Cascade superfusion

Three water-jacketed and thermostatted superfusion chambers were mounted in series [19] and the configuration is outlined in Figure S1. The top chamber, with donor tissue, was preceded by a warming coil through which aerated (5% CO_2_ in O_2_) Tyrode’s solution was pumped at 1.5 mL min^−1^ by means of a peristaltic pump. The fluid was led onto the tissues by the suspending cotton ligature. The donor tissue was connected at 20 mN to an isometric transducer (FT03, Grass Technologies, Warwick, RI, USA) whereas in the following chambers assay ureters were mounted by suspending ligatures connected to Harvard isotonic transducers at 2 mN (Harvard Apparatus, Holliston, MA). The distance between each two chambers was 20 cm and transit time between chambers was approximately 3 s. Muscular activity was recorded with Acknowledge software using a MP100 digitization unit (Biopac Systems Inc., Goleta, CA).

Carbachol could be introduced either by direct syringe injection by hand onto tissues (“direct rapid injection”) or by infusion (Perfusor syringe pumps, B Braun, Melsungen, Germany) into the Tyrode’s solution flow just before the warming coil supplying the donor chamber. By constant infusion at the bottom of the donor chamber using another syringe pump (B Braun), compounds (such as scopolamine) could be directly applied onto assay tissues, thus bypassing the donor tissue.

### NO/nitrite release

Aliquots (1 mL min^−1^) of superfusate, containing L-arginine 10 µM, were collected immediately after the donor chamber and were analysed for NO/nitrite content by immediate injection into a reflux system for NO/nitrite analysis with chemiluminescence detection [22].

### Urothelium staining

After experiments the whole preparation of urothelium-intact and -denuded ureters or urinary bladders were incubated in Tris-HCl buffer solution (50 mM, pH 8) containing 1 mM β-NADPH, 0.5 mM nitroblue tetrazolium and 0.2% Triton X-100 at 37°C for 10 min [23], [24]. After wash in saline tissues were immediately subjected to microscopic observation in reflective light.

### Experimental protocol

After equilibration, carbachol (1–5 µM) was applied to urothelium-intact and -denuded ureters directly. Thereafter scopolamine was introduced in stepwise increasing concentrations to the assay tissues to desired final concentration (5–30 µM), sufficient to block all the effects of carbachol on the ureters. Comparisons of the carbachol applications bypassing or over the donor bladder were studied at equal injection volumes or infusion rates. Both urothelium-intact and -denuded urinary bladders were used as donor tissues under the same conditions and were assayed on urothelium-denuded ureters. Subsequently, the nitric oxide synthase inhibitor *N^G^*-nitro-L-arginine methyl ester (L-NAME) (100 µM), the adenosine/P1 nucleoside receptor antagonist 8-(*p*-sulfophenyl)theophylline (8-PST) (100 µM) or the cyclo-oxygenase inhibitor diclofenac (1 µM) was added into the superfusion reservoir separately. After donorand assay tissues were exposed to these blocking agents for at least 30 min, the carbachol applications were repeated. A flow chart (Figure S2) of these experiments is found in Supporting Information. In experiments on NO/nitrite release from bladders, basal release and release during infusion of acetylcholine 100 µM in the absence and presence of 0.3 µM tetrodotoxin or 300 µM L-NAME were compared. Tetrodotoxin was used to estimate how much of NO/nitrite release was dependent on nerve activation. The L-NAME concentration used was to overcome the added 10 µM L-arginine which had to be included in the NO/nitrite release experiment in order to maintain a stable release [22].

### Drugs

Sodium pentobarbital was purchased from Apoteksbolaget, Stockholm, Sweden. Carbachol, scopolamine, L-NAME (*N^G^*-nitro-L-arginine methyl ester), diclofenac, β-NADPH, nitroblue tetrazolium and routine chemicals were from Sigma-Aldrich Chemical Co, St Louis, MO, USA. 8-PST (8-(*p*-sulfophenyl)theophylline) was from Sigma and was in addition synthesized as previously described [15].

### Statistical analysis

Frequency of ureteral spontaneous contractions were expressed as beats per minute, and was determined by use of the Find Rate and Mean Value functions of the Acknowledge software. Data was expressed as means ± SEM. Results from ureter tissues which initially showed maintained contractile frequency below 0.3 beats per min (BPM) were discarded. Statistical significance was analyzed by Student’s *t*-test for paired data or by ANOVA for several groups or repeated measures, as appropriate. Significance was considered at *P*<0.05.

## Results

**Figure 1:**
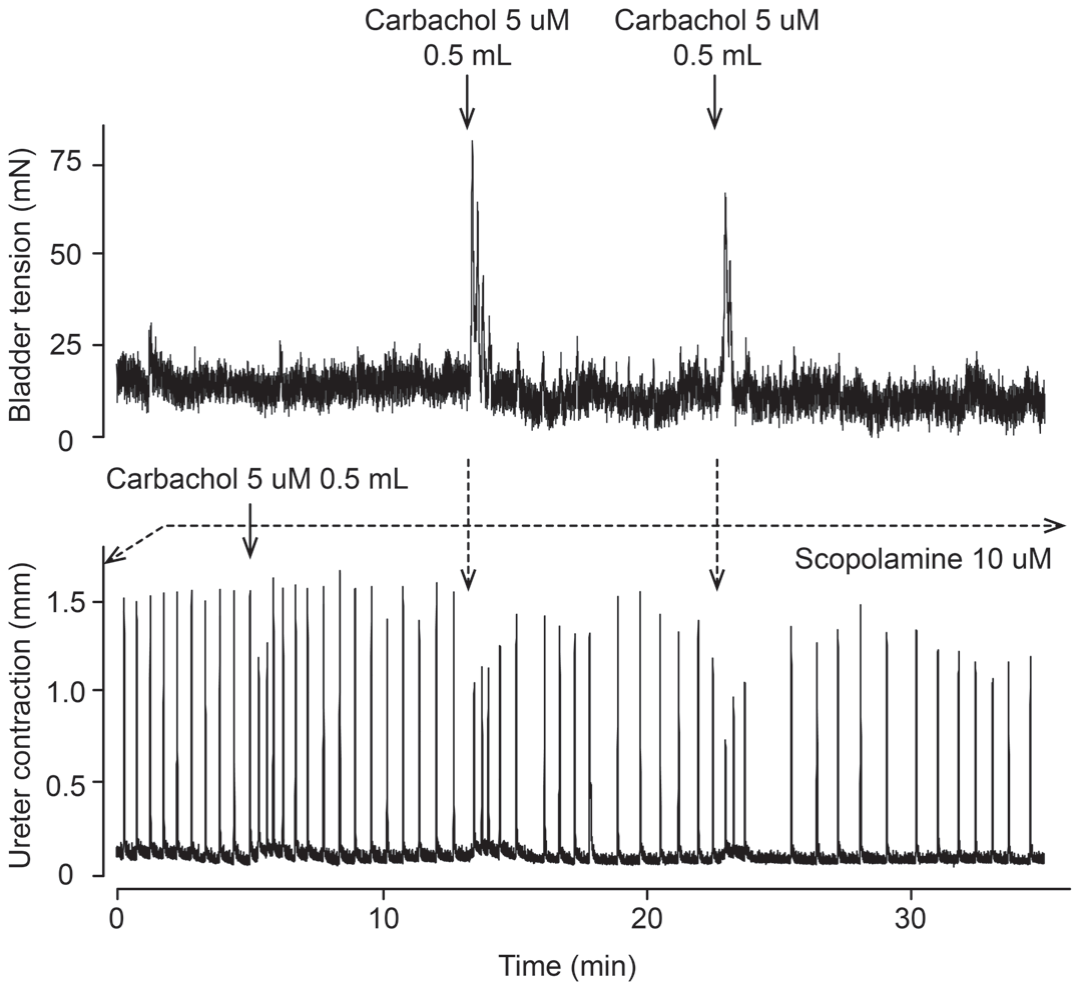
Experimental recording of contractions of an everted urothelium intact guinea pig urinary bladder (top tracing) and an assay urothelium-denuded guinea pig ureter (lower panel) in serial superfusion mode. Solid arrows indicate injection of 0.5 µM final concentration in superfusate flow (1.5 mL per min) to assay ureter where the injection either bypassed the bladder by injection into the flow below the donor bladder (lower solid arrow) or via injection before the bladder (top solid arrows). Scopolamine 10 µM was administered to assay ureter throughout.

Guinea pig ureters exhibited regular spontaneous contractions when superfused by Tyrode’s solution at 1.5 mL min^−1^. The rapid spontaneous phasic contractions (Figure 1, lower panel) usually occurring at a rate of 0.5–1.5 beats per minute were relatively stable over 1–2 hours and then declined in frequency and amplitude, a phenomenon more prominent in urothelium-intact ureters. When infusing 1 µM carbachol directly onto ureters, the urothelium-intact ureters showed inhibition of contractile frequency after a short burst of increased beats, while in the urothelium-denuded ureters carbachol exerted only excitatory effects, without any ensuing decrease in contraction rate (see Figure S3). Nearly all of the excitatory effects by direct rapid injections of carbachol onto denuded ureters could be blocked by continuously infusing scopolamine 10 µM into the superfusing fluid. However, at times and probably due to high peak concentrations of carbachol with the injection technique an excitation could still be seen (Figure 1). Thus, after brief applications of 5 µM carbachol (0.5 mL in a 1.5 mL per min flow) directly to scopolamine-blocked urothelium-denuded ureters, only excitatory effects were seen, whereas the same amount of carbachol injected over the urothelium-intact bladder, subsequently reaching the ureter, showed significant inhibition of assay ureter contractions, sometimes preceded by an initial excitation (Figure 1). The inhibitory effect was reproducible by repeated injections of carbachol and lasted several minutes (Figure 1). The second assay ureter usually exhibited irregular phasic contractions, and it was therefore difficult to establish whether the inhibitory activity was transmitted over the 6 s delay to this tissue.

**Figure 2:**
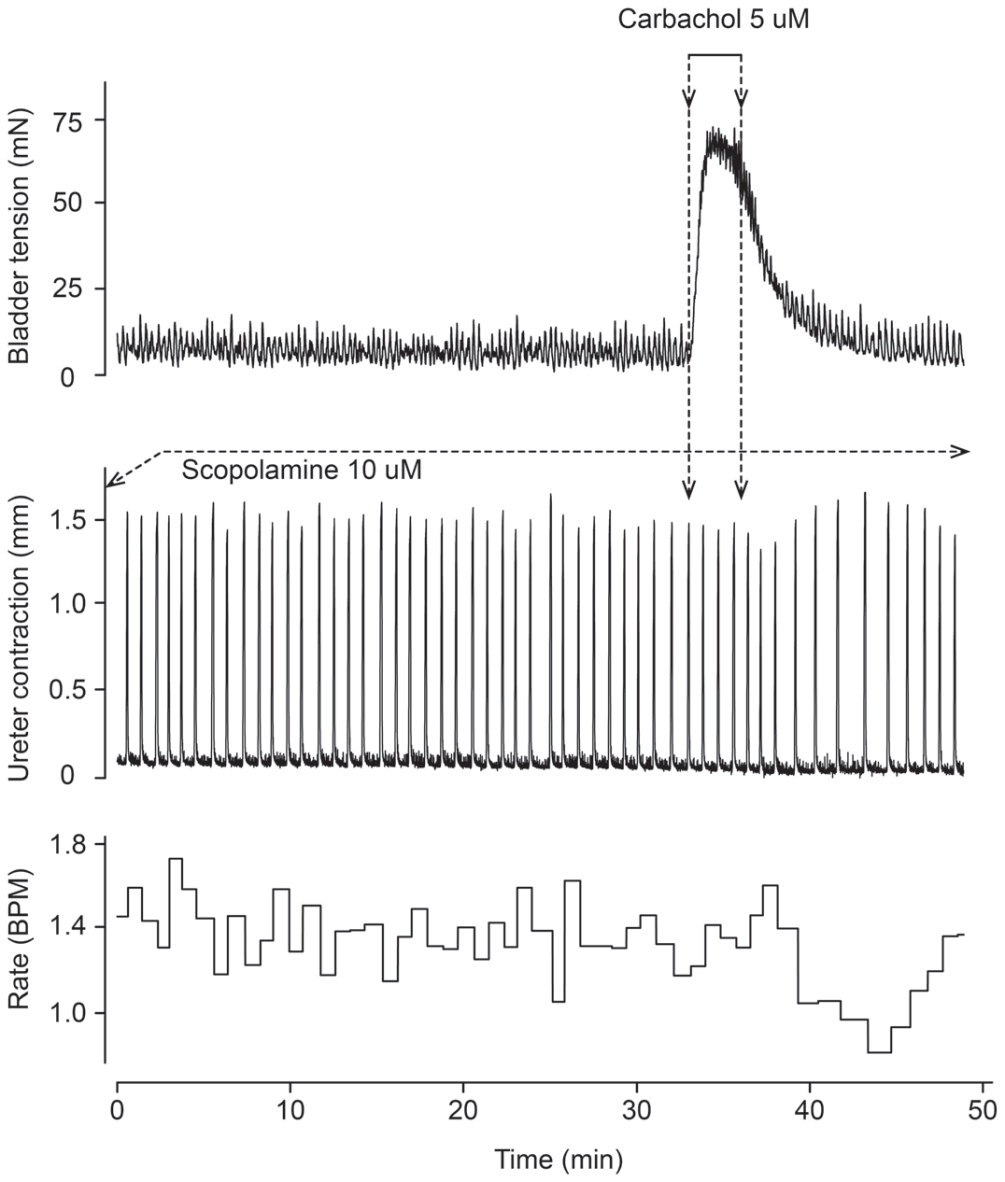
Experimental recording of contractions of an everted urothelium-intact guinea pig urinary bladder (top tracing) and an assay urothelium-denuded guinea pig ureter (middle tracing) in serial superfusion mode, showing the effects of a prolonged (2 min) administration of carbachol 5 µM to the donor tissue by infusion at the top of the cascade system. The bottom panel shows a computerized analysis of the spontaneous contraction frequency of the assay ureter (Biopac Acknowledge software). Scopolamine 10 µM was administered to the assay ureter throughout. Carbachol administered before the urothelium-intact donor bladder caused a minor drop in basal tone of the assay ureter, and a delayed-in-onset and prolonged inhibition of spontaneous contractions in the assay ureter.

**Figure 3:**
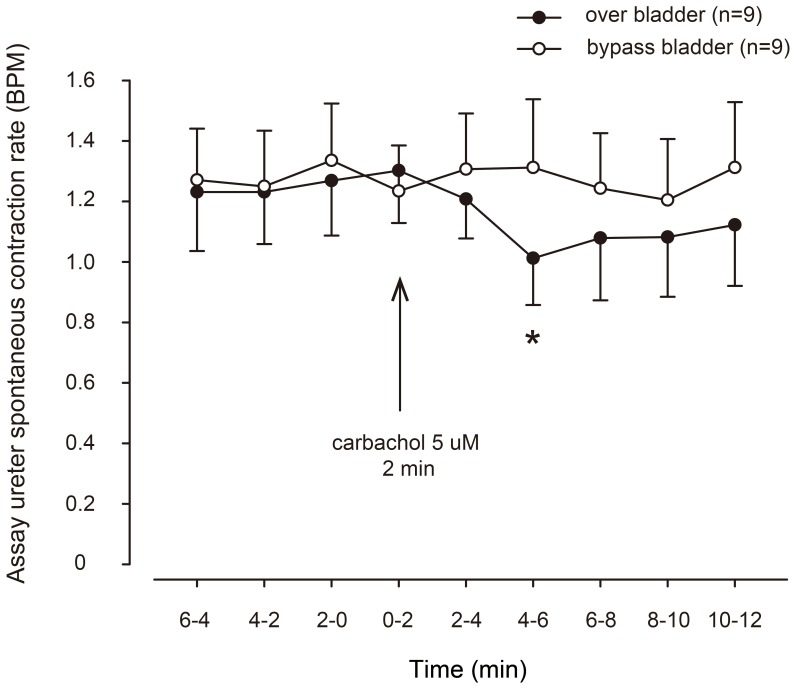
Time course of transmissible bioactivity released from urothelium-intact guinea pig urinary bladders, determined as change in spontaneous contraction frequency of assay ureters in serial superfusion. Comparison is made between administration of carbachol before the bladder (solid symbols) or administered after the bladder but above the assay ureter (open symbols). *denotes p<0.05 by repeated measures ANOVA. n = 9, n denotes number of animals.

Since the method of direct rapid injection likely entails the risk of high and variable carbachol concentrations, and also the possibility of cooling effects contributing to the observed inhibitory effects, 2 min constant rate infusions of carbachol (with purportedly more well-defined concentrations of agonist in the tissue) were made through the prewarming coil onto urothelium-intact urinary bladders, and were compared with direct rapid injection of carbachol immediately before the assay ureters (Figure 2). Similar prolonged inhibitory effects as with the direct rapid injection experiments were obtained in the first assay ureter, during and after the now prolonged contraction of the donor tissue. The excitatory effects when the infused superfusate reached the assay ureter were essentially absent. The inhibitory effects manifested either as decreasing contractile frequency or combination of initially decreased frequency and lower amplitude together with a minor basal tone decline. The decrease in frequency was sometimes accompanied by an increase in amplitude of contractions (Figure 2). No consistent pattern in the amplitude changes could be found, however, and therefore the statistical evaluation of the responses was performed by computerized analysis of frequency changes in assay ureter contractions. In the computerized evaluation of inhibitory effects the time course was confirmed to be slow, the maximal drop in contraction frequency occurring at 4–6 min after commencing the 2 min carbachol infusion (Figure 3). For the remainder of the cascade experiments the infusion technique was employed to ensure stable concentrations of carbachol to avoid the risk of breakthrough of the scopolamine blockade as evidenced by the excitatory effects in Figure 1.

**Figure 4:**
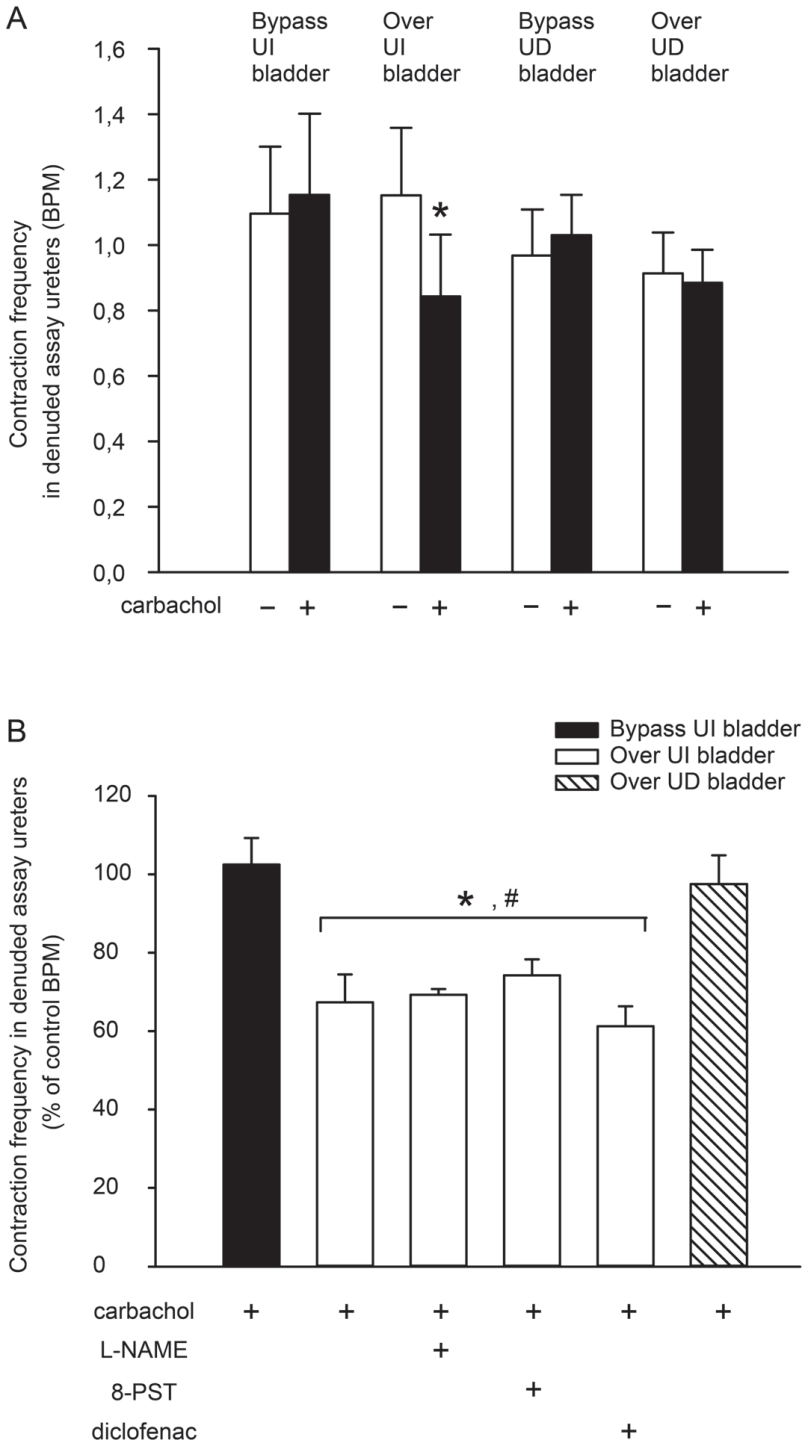
Summary of carbachol induced release of urothelium-derived inhibitory activity from guinea pig urinary bladders bioassayed on ensuing urothelium-denuded ureters superfused in series, by determination of the ureter spontaneous contraction frequency in the absence of (−) or following (+) carbachol administration to the superfusate. *Panel A:* Open columns denote the assay ureter contraction frequency before carbachol and filled columns denote the contraction frequency at 4 min after carbachol, the time point for maximal expected effect as shown in Figure 3. Carbachol was either administered before (“Over”) or after (“Bypass”) the donor tissue which was either urothelium-intact (“UI”) or urothelium-denuded (“UD”). **denotes p<0.01 by Student’s t-test for paired data. Each treatment group contained 8 animals. *Panel B:* Assay ureter contraction frequency at 4 min after the administration of carbachol either before (“Over”) or after (“Bypass”) the donor urinary bladder tissue, which was either urothelium-intact (“UI”) or urothelium-denuded (“UD”). The contractile frequency was expressed in percentage of the contraction frequency determined during 10 min before the application of carbachol. The open columns show the effect of carbachol in the absence and presence of either of either L-NAME (100 µM), 8-PST (100 µM) or diclofenac (1 µM). *denotes p<0.05 for all carbachol applications before (“Over”) in comparison with carbachol application after (“Bypass”) the donor tissue in the absence and presence of drug treatments. # denotes no significant difference between antagonist/inhibitor treatments when compared against each other and against carbachol alone, all applied before (Over) the tissue. Comparisons were made by repeated measures ANOVA. Each treatment group contained 8 animals.

In order to investigate whether the observed transmissible inhibitory activity was emanating from the bladder wall or from the urothelium, experiments comparing carbachol-induced bioactivities from urothelium-intact and urothelium-denuded bladders were performed (Figure 4A). Comparisons were made with effects of carbachol applied directly to the scopolamine-treated assay ureters, thus bypassing the bladder tissue. These experiments showed that an inhibitory effect could only be seen when carbachol was administered over urothelium-intact donor urinary bladders (Figure 4A).

Besides being well known inhibitors in the urinary tract [13], [14], [25]–[27] adenosine and nitric oxide exert inhibitory actions on smooth muscle in many other systems. Prostaglandins may have several functions in the urinary tract, where they can inhibit the peristalsis of ureters and may also be very important in maintaining spontaneous activity of the ureter [28]. We investigated if blocking these mediators could abolish the urothelium-dependent transmissible bioactivity. L-NAME, 8-PST or diclofenac were added into the superfusion reservoir separately, and subsequently urothelium-intact donor bladders were challenged again with carbachol. The treatments had a tendency of slightly lowering the spontaneous contractile frequency of the ureters, but the effects of carbachol infusions remained. Thus, the contraction frequency of assay ureters were still inhibited by transmissible inhibitory effects when carbachol was infused over urothelium-intact bladders in the L-NAME, 8-PST and diclofenac pre-treated groups (Figure 4B).

**Figure 5:**
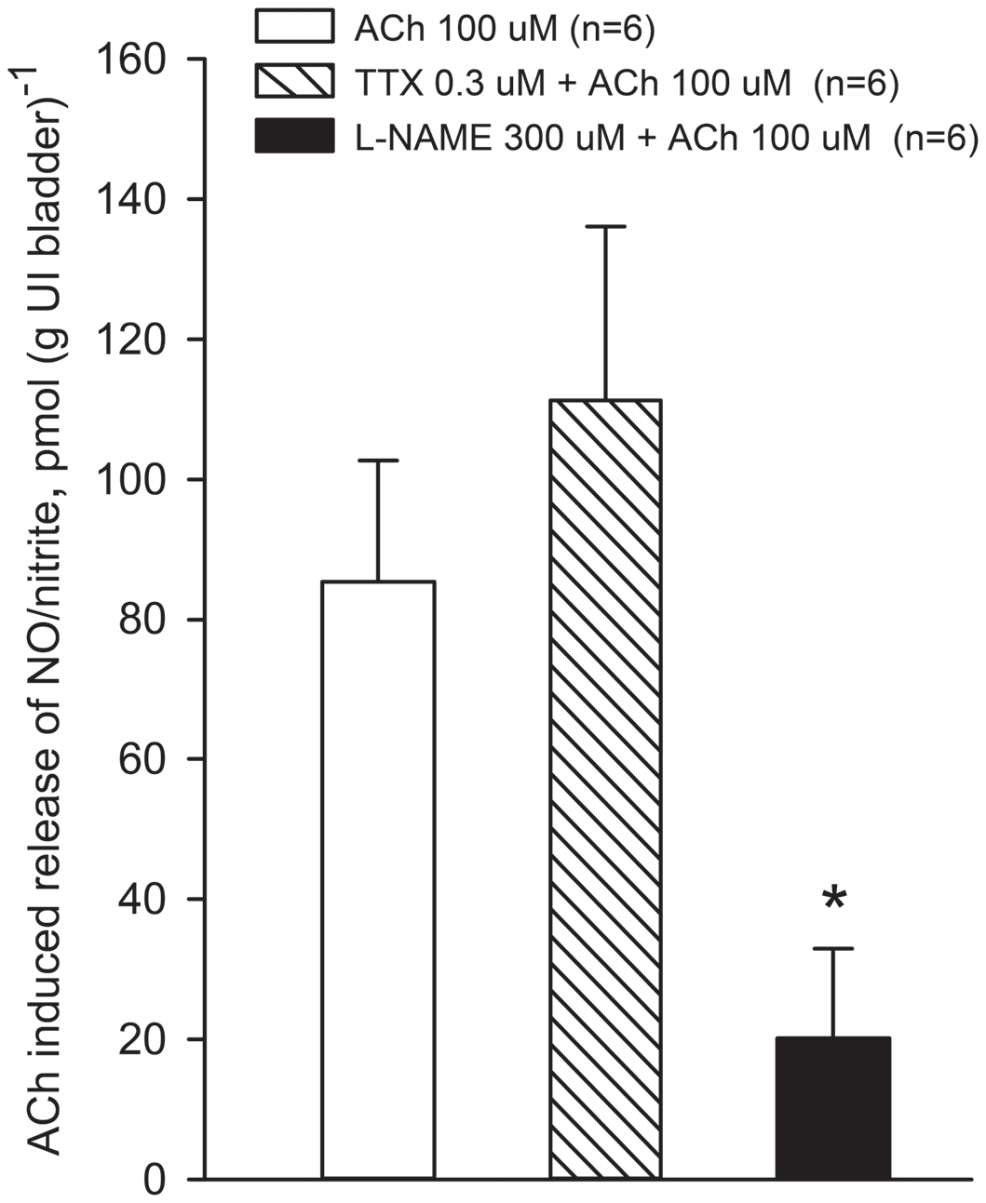
Acetylcholine-evoked NO/nitrite release from isolated superfused urothelium-intact (UI) guinea pig urinary bladders, determined by chemiluminescence detection after injection of superfusate fractions into a reflux system for nitrite reduction (see Methods). Acetylcholine was applied either alone (open column) or in the presence of tetrodotoxin (TTX) (hatched column) or L-NAME in the superfusion fluid (filled column). *denotes p<0.05 for the L-NAME group versus either acetylcholine alone or in the presence of tetrodotoxin as determined by one-way ANOVA on multiple groups. n = 6, n denotes number of animals.

NO/nitrite release from urothelium-intact donor bladders was measured before and during application of L-NAME, which was found to inhibit the release by more than 75% (Figure 5). This was despite the fact that L-arginine had to be included in the superfusate to maintain a reproducible release of NO/nitrite. The sodium channel blocker tetrodotoxin did not alter NO/nitrite release.

**Figure 6:**
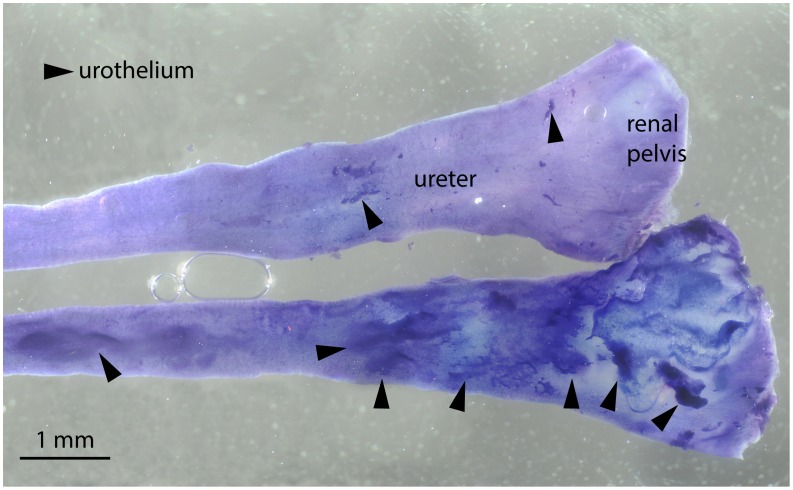
NADPH-diaphorase staining of two guinea pig ureters stained together after a cascade superfusion experiment. Ureters were opened longitudinally before the experiment and are shown with their originally internal side facing upwards towards the viewer. Top tissue was denuded from as much urothelium as possible before start of experiment. Urothelium was stained dark blue by the diaphorase reaction (bottom tissue, and some small specks in top tissue), for clarity indicated by filled arrow-heads. Some urothelium fell off from urothelium intact ureter (lower tissue, left part), but was still present on the majority of the original internal surface. Small pieces of urothelium remaining in the top urothelium denuded ureter, as indicated. Multiple photographs were obtained in incident light in a Zeiss Laboratory Standard 16 microscope with Zeiss F 2.5/0.08 objective, and merged by ZMcombine software (freeware). Nikon D300 camera with an f = 350 mm adapter (Wild 308797) using Breeze Systems Ltd (Bagshot, Surrey, UK) D300Remote image capture software.

To confirm the removal of urothelium from ureters and bladders, NADPH-diaphorase staining and microscopy was carried out directly after experiments. Several staining techniques were investigated but yielded poor or no staining of the urothelium whereas the NADPH diaphorase reaction exhibited prominent staining of the urothelium (Figure 6). The difference between urothelium-covered and urothelium-denuded areas was clearly visible, allowing confirmation of successful urothelium removal in urothelium-denuded bladders and ureters.

## Discussion

The three main findings of the present study are that denuded guinea pig ureters can be used in serial superfusion to bioassay released inhibitory bioactivity from the guinea pig urinary bladder, and that such release is urothelium-dependent and is transmissible over a significant distance. This, in our opinion, should open up the possibility of attempting isolation of the elusive urothelium-derived relaxing factor.

In vitro isolated ureters have long been used for studies on urinary tract motility, since they can exhibit spontaneous rhythmic activity, much in analogy with the rhythmic ureteral peristalsis seen in vivo. These myogenic rhythmic contractions of the upper urinary tract are triggered by pacemaker cells located in the renal pelvis and conducted through the whole ureter by atypical smooth muscle cells [6]. In our experiments, scopolamine did not modify the ureter spontaneous contractions suggesting that scopolamine is a suitable blocker for the cholinergic agonists used for stimulating donor tissues mounted above the assay ureters and supporting the idea that the spontaneous contractions are independent of intrinsic cholinergic activity in the ureter tissue. Compared with other possible assay tissues, such as aorta [5], [18]–[20], ureter might share more similarities with bladder muscle in receptor subtypes and binding affinity. Therefore it seems natural to use ureter as assay tissue to study the released factors from urinary bladder, giving ample possibility for direct action onto the ureter smooth muscle. The second assay ureter tissue in our serial superfusion system did not exhibit sufficiently regular contractions comparable with the first one in the serial superfusion, and did not allow conclusion of transmissible factor to this tissue. One complication might be successively lower oxygen supply down the cascade. Also, the lower assay tissue received compounds released from both the donor bladder and upper assay ureter, whereby the released factors might act to desensitise or inhibit the second assay ureter or causing it not to respond regularly and sustainably.

As mentioned before, several studies showed that in the presence of urothelium, the contractile responses of isolated urinary bladder strips in different species in response to many stimulators were smaller compared with urothelium-denuded bladder strips [2], [3]. The smaller responses in such strips might be due to poor agonist penetration through urothelium into smooth muscles, or alternatively that inhibitory factor was released from urothelium as proposed in several studies. By using urothelium-intact and -denuded donor bladder tissue, and assaying on ureters in our experiments, we could ascertain that the inhibitory effect seen on assay ureters was coming from bladder urothelium. That merely the mechanical contraction of the donor bladder was a cause for the release of inhibitory bioactivity seems unlikely since, in a previous study, stimulating the bladder with α-adrenoceptor agonist failed to release inhibitory factor although it induced significant contraction of the bladder tissue [16]. High concentration of KCl and neurokinin A evoked contractile responses on human detrusor which were not affected by urothelium removal [17]. We therefore believe that released inhibitory activity is not simply a reflection of direct bladder detrusor muscle contraction, but seems to be a more complicated process involving muscarinic receptor activation and where urothelium is a key component in this process.

Nitric oxide is released from bladder urothelium [29], [30], and can relax bladder smooth muscle where urothelium-derived NO has been considered having a role in regulating detrusor muscle function [31]. ATP and adenosine can inhibit nerve induced contractile responses in rat urinary bladder [13] and are important regulators of bladder function [7], [32]. In our experiments, the nitric oxide synthase inhibitor L-NAME and the adenosine receptor blocker 8-PST did not modify the transmissible inhibitory activity, and L-NAME caused a marked decrease in the release of NO/nitrite. Nitric oxide, its metabolite nitrite, or a P1 purinoceptor agonist like adenosine are therefore unlikely to be the unknown factor or involved in the release of the factor from urothelium. Further studies are still needed to exclude other purines such as ATP and related nucleotides, due to their significant release from bladder urothelium [8], [9], and since ATP in some species can inhibit or relax the bladder [33]–[35]. Judged from our own experiments, ATP seems an unlikely candidate in the guinea pig since here it is contractile on the ureter [27]. Prostaglandins and prostacyclin are known to be synthesized by the cyclo-oxygenase localized in the urothelium which modulate the contractions of the urinary tract [6]. Although, presently, the cyclo-oxygenase inhibitor diclofenac did not abolish the carbachol induced transmissible inhibitory activity, prostanoids seem to play important roles in the modulation of urinary tract motility. It was proposed that the spontaneous motility of urinary tract depends on local release and balance of both excitatory and inhibitory prostanoids [12]. More investigations concerning prostanoids and whether they might constitute at least part of the inhibitory factor being released from urinary bladder seem to be motivated, especially since it has been reported to be difficult to fully inhibit the release of prostanoids from urothelium-containing bladder tissue by application of a cyclo-oxygenase inhibitor [36].

This is the first study to show that the inhibitory biological activity released from urinary bladder urothelium is transmissible from one bath to another, which is a considerable distance compared with previous sandwich models. This will in the cascade superfusion technique (Figure S1) allow further pharmacological analysis with blockers or other modifiers, in addition to the presently used, since such modifiers can be added not only jointly but now also separately between donor and assay tissues. The technique in future experiments also allows use of different bioassay tissues for differential bioassay or introduction of capturing material or other physical means in the superfusion flow, when aiming at chemical characterisation of the bioactive principle or principles. We thus noticed that the urothelium-derived factor not only inhibited the contractile frequency but also caused a decline in the basal tone of the assay ureter. This is in some agreement with results from a previous study from the Iselin group where removal of the urothelium of ureters made stimulants evoke both phasic and tonic increase of ureter motility [12]. Such a suppressive effect might be exerted by a single compound released from the urothelium acting via different receptors or there could exist several excitators and inhibitors in the superfusion fluid which in a more complex fashion lead to inhibition of the ureter motility. The presently observed maximal effect in suppression of phasic ureter contractions ocurred at around 4 minutes after carbachol application and was maintained about 2 minutes. If caused by a single autacoid, the inhibitor does not seem to be a rapid mediator. This quality might suggest favourable conditions with the present technique, for attempts with further characterisation and isolation. Identification of the principle would greatly enhance the understanding of overactive bladder syndrome and facilitate attempts at finding novel therapeutic approaches of this type of debilitating condition [37], [38]. In future studies ATP and other nucleotides should be considered since ATP has been shown to exert not only excitatory but also inhibitory effects in bladder tissue [33]–[35].

In summary, it has been shown previously by use of sandwich-type experiments that a urothelium-derived relaxing activity is transmissible over a short distance. The present report shows that the urothelium-derived activity is not a fast reacting activity and can be transferred over a considerable distance, and thus might be amenable for isolation and identification. The identity of the urothelium-derived relaxing factor is not known and the mechanisms underlying its release are not known, but the present data suggest that the inhibitory factor is not nitric oxide or an adenosine receptor agonist. Although we obtained indirect evidence that it is not a cyclo-oxygenase product this must be interpreted with caution due to known difficulties in inhibiting urothelium-dependent prostaglandin generation. Further studies are needed on the roles of cyclo-oxygenase products in the modulation of release and function of urothelium-derived relaxing factor and to clarify the nature of the unknown compound(s).

## Supporting Information

Figure S1 **Cascade superfusion setup.** Donor tissue was guinea pig spirally cut whole urinary bladder with or without urothelium. Assay tissues were guinea pig ureters. Infusion pump denotes where one or several infusion pumps were connected for administration of agonists or blockers. Modified from Gryglewski et al., 1986.
(PDF)

Figure S2 **Flowcharts for experimental procedures.** Upper panel illustrates a control experiment where 3 min infusions of the agonist carbachol were performed in the absence of blockers on the donor tissue, but where scopolamine was infused to prevent an effect of carbachol on the assay ureter. Lower panel illustrates similar experiments where either of the indicated blockers were administered.
(PDF)

Figure S3 **Experimental recordings of isolated and separately superfused guinea pig ureters.** Spontaneous contractions recorded isotonically. Top panel: urothelium-intact (UI) ureter. Bottom panel: urothelium-denuded (UD) ureter. Carbachol was infused for 3 min into the superfusion fluid above the ureters as indicated, evoking early increase in contraction frequency followed by inhibition in the urothelium-intact ureter, whereas only excitation was seen in the urothelium-denuded ureter. Scoplolamine was not present in this experiment.
(PDF)
