# Supplementary figures and images for: Cascade Bioassay Evidence for the Existence of Urothelium-Derived Inhibitory Factor in Guinea Pig Urinary Bladder
Source: PLoS One. 2014 Aug 1;9(8):e103932. doi: 10.1371/journal.pone.0103932 (PMC4118989; doi:10.1371/journal.pone.0103932)

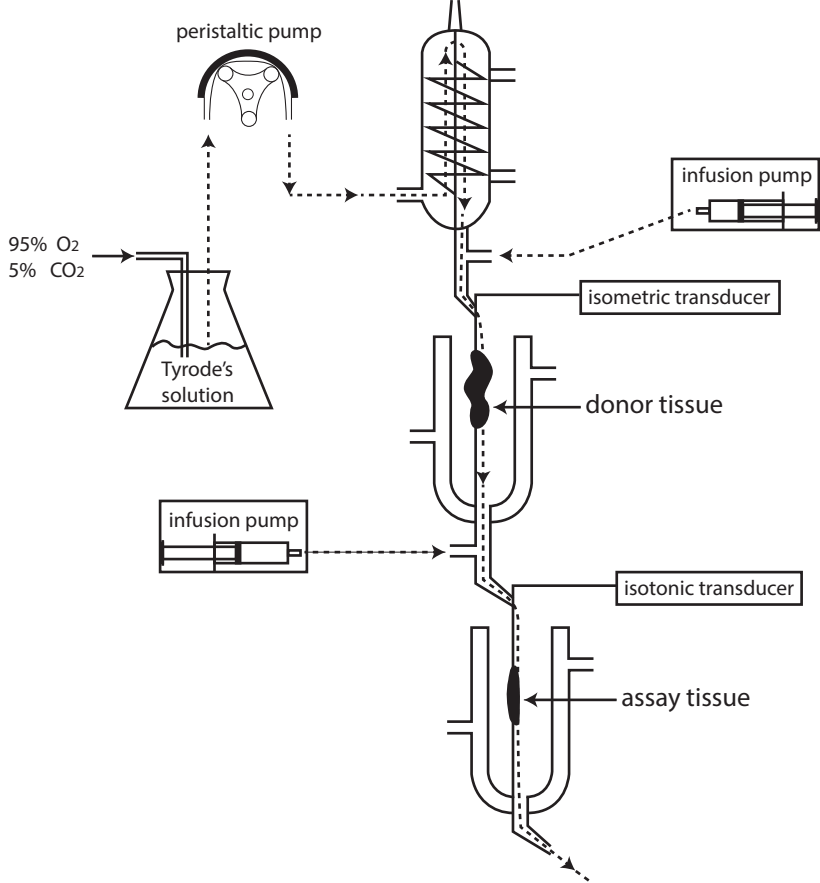

Supplement: Figure S1 — Cascade superfusion setup. Donor tissue was guinea pig spirally cut whole urinary bladder with or without urothelium. Assay tissues were guinea pig ureters. Infusion pump denotes where one or several infusion pumps were connected for administration of agonists or blockers. Modified from Gryglewski et al., 1986. (PDF) [file pone.0103932.s001.pdf]

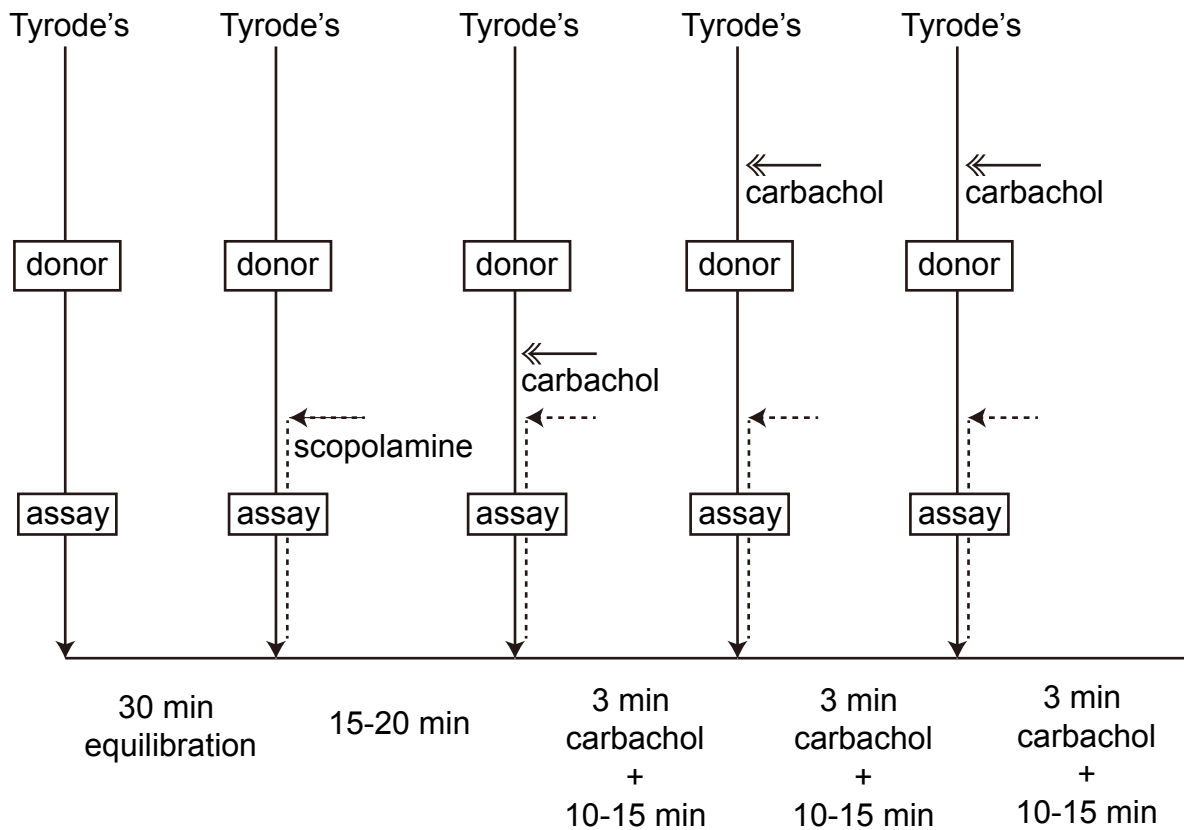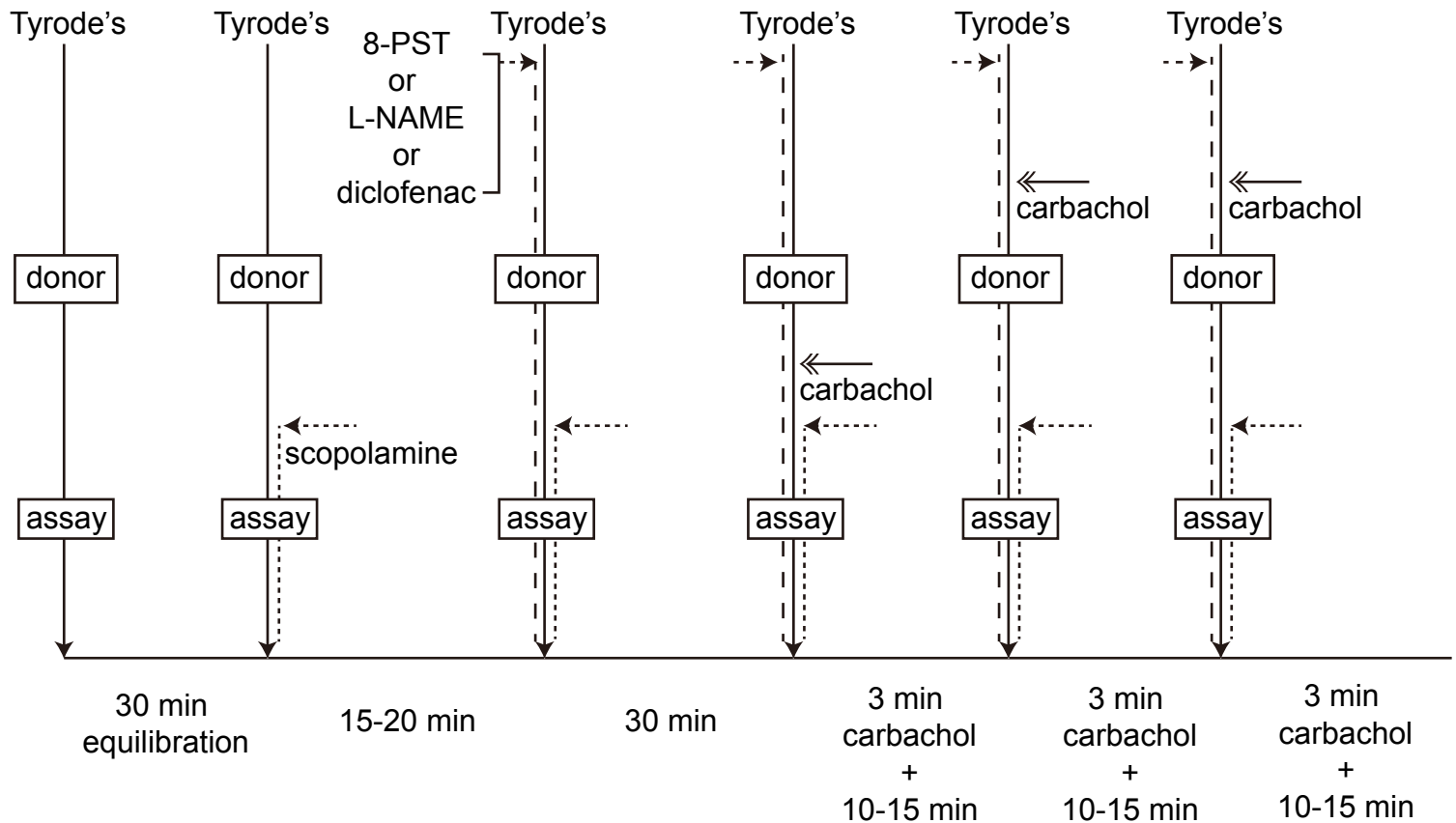

Supplement: Figure S2 — Flowcharts for experimental procedures. Upper panel illustrates a control experiment where 3 min infusions of the agonist carbachol were performed in the absence of blockers on the donor tissue, but where scopolamine was infused to prevent an effect of carbachol on the assay ureter. Lower panel illustrates similar experiments where either of the indicated blockers were administered. (PDF) [file pone.0103932.s002.pdf]

carbachol 1  $\mu$ M

Guinea pig  
UI ureter

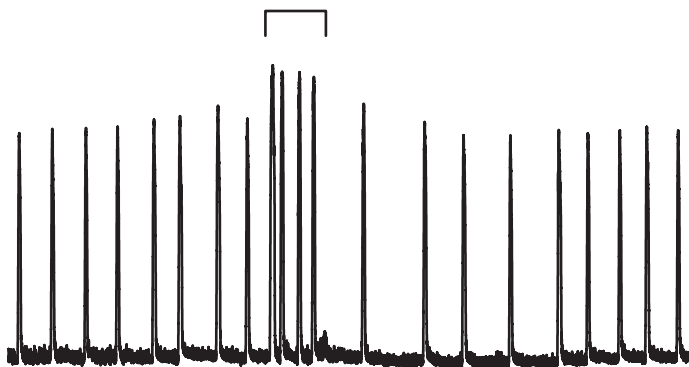

0.4 mm

3 min

carbachol 1  $\mu$ M

Guinea pig  
UD ureter

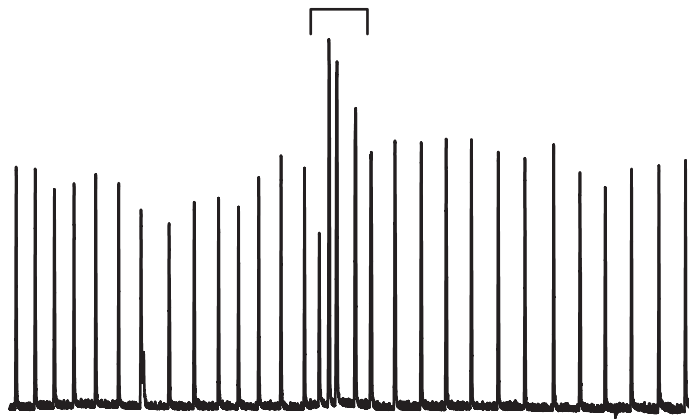

0.5 mm

3 min

Supplement: Figure S3 — Experimental recordings of isolated and separately superfused guinea pig ureters. Spontaneous contractions recorded isotonically. Top panel: urothelium-intact (UI) ureter. Bottom panel: urothelium-denuded (UD) ureter. Carbachol was infused for 3 min into the superfusion fluid above the ureters as indicated, evoking early increase in contraction frequency followed by inhibition in the urothelium-intact ureter, whereas only excitation was seen in the urothelium-denuded ureter. Scoplolamine was not present in this experiment. (PDF) [file pone.0103932.s003.pdf]
